# TLR4 mutation protects neurovascular function and cognitive decline in high-fat diet-fed mice

**DOI:** 10.1186/s12974-022-02465-3

**Published:** 2022-04-29

**Authors:** Nathalie Obadia, Giulia Andrade, Marina Leardini-Tristão, Letícia Albuquerque, Celina Garcia, Flavia Lima, Júlio Daleprane, Hugo C. Castro-Faria-Neto, Eduardo Tibiriçá, Vanessa Estato

**Affiliations:** 1 Laboratory of Immunopharmacology, Oswaldo Cruz Institute, FIOCRUZ, Av. Brasil, 4365, Manguinhos, Rio de Janeiro, 21040-900 Brazil; 2 Laboratory of Glial Cell Biology, Institute of Biomedical Sciences, Federal University of Rio de Janeiro, Rio de Janeiro, Brazil; 3 Interdisciplinary Nutrition Assessment Laboratory, Rio de Janeiro State University, Rio de Janeiro, Brazil; 4 National Institute of Cardiology, Rio de Janeiro, Brazil

**Keywords:** Brain microcirculation, High-fat diet consumption, TLR4, Neuroinflammation, glial cells

## Abstract

**Background:**

Metabolic syndrome (MS) is defined as a low-grade proinflammatory state in which abnormal metabolic and cardiovascular factors increase the risk of developing cardiovascular disease and neuroinflammation. Events, such as the accumulation of visceral adipose tissue, increased plasma concentrations of free fatty acids, tissue hypoxia, and sympathetic hyperactivity in MS may contribute to the direct or indirect activation of Toll-like receptors (TLRs), specifically TLR4, which is thought to be a major component of this syndrome. Activation of the innate immune response via TLR4 may contribute to this state of chronic inflammation and may be related to the neuroinflammation and neurodegeneration observed in MS. In this study, we investigated the role of TLR4 in the brain microcirculation and in the cognitive performance of high-fat diet (HFD)-induced MS mice.

**Methods:**

Wild-type (C3H/He) and TLR4 mutant (C3H/HeJ) mice were maintained under a normal diet (ND) or a HFD for 24 weeks. Intravital video-microscopy was used to investigate the functional capillary density, endothelial function, and endothelial–leukocyte interactions in the brain microcirculation. Plasma concentrations of monocyte chemoattractant protein-1 (MCP-1), adipokines and metabolic hormones were measured with a multiplex immunoassay. Brain postsynaptic density protein-95 and synaptophysin were evaluated by western blotting; astrocytic coverage of the vessels, microglial activation and structural capillary density were evaluated by immunohistochemistry.

**Results:**

The HFD-induced MS model leads to metabolic, hemodynamic, and microcirculatory alterations, as evidenced by capillary rarefaction, increased rolling and leukocyte adhesion in postcapillary venules, endothelial dysfunction, and less coverage of astrocytes in the vessels, which are directly related to cognitive decline and neuroinflammation. The same model of MS reproduced in mice deficient for TLR4 because of a genetic mutation does not generate such changes. Furthermore, the comparison of wild-type mice fed a HFD and a normolipid diet revealed differences in inflammation in the cerebral microcirculation, possibly related to lower TLR4 activation.

**Conclusions:**

Our results demonstrate that TLR4 is involved in the microvascular dysfunction and neuroinflammation associated with HFD-induced MS and possibly has a causal role in the development of cognitive decline.

**Supplementary Information:**

The online version contains supplementary material available at 10.1186/s12974-022-02465-3.

## Background

Over the past two decades, studies to determine a mechanism that links the pathogenesis of obesity to insulin resistance and diabetes have revealed a close association between nutrient excess and innate immune system activation [1]. Understanding the pathophysiology and risk factors for a range of chronic diseases with the precursor concept of inflammation could help treat associated comorbidities. Metabolic syndrome (MS) is defined as a combination of risk factors that culminate in adverse outcomes [2, 3], including type 2 diabetes mellitus, cardiovascular disease [4, 5], and neurodegenerative diseases [6]. At least three risk factors are associated with MS: obesity in conjunction with hyperglycemia, high arterial blood pressure, and dyslipidemia, usually associated with hyperinsulinemia and insulin resistance [7–10]. Overnutrition is an environmental stimulus that can activate Toll-like receptor (TLR) pathways to mediate the development of MS-related disorders [11, 12].

There is evidence that the combination of obesity and hypertension can impair performance across various cognitive domains [13]. In addition, type 2 diabetes mellitus constitutes a strong risk factor for cognitive decline and Alzheimer’s disease, as well as generally poorer memory performance in adults [14–16]. Animal studies have shown that a high-calorie diet impairs the structure and function of the hippocampus and cortex, critical brain regions for learning and memory [17]. The main factor conferring a risk of cognitive decline in obese and hyperglycemic individuals is neuroinflammation, which is characterized by the activation of the resident population of macrophages and microglia, initiating/priming an inflammatory state that leads to the establishment of low-grade neuroinflammation [18, 19].

The neurovascular unit (NVU), formed by neurons, interneurons, astrocytes, and basal lamina covered with smooth muscle cells, pericytes, endothelial cells, and extracellular matrix, enables cross-talk between neurons and microcirculation [20]. Each component is closely and reciprocally linked, establishing an anatomical and functional role, which results in a highly efficient system for regulating cerebral blood flow [21].

It has been proposed that changes in the NVU lead to brain dysfunction and damage, and there is a growing interest in exploring the potential contribution of neurovascular dysfunction to neurodegeneration [22, 23]. However, the mechanistic link between changes in the NVU components and cognitive decline in obese experimental models is not yet fully understood.

In the present study, we investigated the role of TLR4 in the brain microcirculation and cognitive performance of diet-induced MS mice. Our objectives were to 1) determine whether mice lacking functional TLR4 would be protected against microvascular brain disorders during high-fat diet (HFD)-induced MS and 2) identify the underlying NVU components, such as astrocytes, blood vessels, neurons, and microglia, that were altered during HFD-induced MS.

## Methods

### Animals and experimental protocol

We used C3H/HeJ and C3H/He mice. The animals were supplied from the central animal facilities of the Oswaldo Cruz Foundation, Brazil, and were housed in standard cages in a temperature-controlled room (22 ± 1 °C) with a 12 h light/dark cycle and water ad libitum. All experimental procedures were conducted in accordance with the internationally accepted principles for the Care and Use of Laboratory Animals and were approved by the Oswaldo Cruz Foundation Animal Welfare Committee (license # 038/2015). C3H/HeJ mice are used in many areas of research, including cancer, immunology, and inflammation, as well as sensory and cardiovascular biology; they have a mutation in the third exon of TLR4, which compromises TLR4 signaling [24, 25]. C3H/He mice were used as background control mice (WT).

At eight weeks of age, the wild-type (C3H/He) and TLR4 mutant (C3H/HeJ) mice were randomly divided into two groups for each mouse strain (n = 12 per group) and fed either standard chow (AIN-93 M; normolipid diet, ND) or a high-fat diet (HFD) for twenty-four weeks. The ND contained 14% protein, 77% carbohydrate, and 9% lipids, and the HFD contained 14% protein, 33% carbohydrate, 53% lipids, and 0.5% NaCl. Both diets were manufactured by Prag Soluções (Jaú, SP, Brazil). All animals were weighed at the beginning and end of the diet period. The groups are referred to as WT-ND, WT-HFD, TLR4-mut-ND, and TLR4-mut-HFD.

### Hemodynamic measurements

Systolic arterial pressure (SAP) and heart rate (HR) were assessed by a noninvasive measurement of tail pressure in conscious animals at the beginning and in the 24th week of the experimental protocol using a computerized tail-cuff plethysmography system (BP-2000 Blood Pressure Analysis System; Visitech Systems, Apex, NC, USA). The animals were adapted to the apparatus for three consecutive days prior to undergoing their baseline measurements.

### Morris water maze

One week before the end of the diet period, cognitive function was investigated by the Morris water maze (MWM) test, which consisted of a black circular pool (100 cm in diameter) that was conceptually divided into four equal, but imaginary, quadrants for the purpose of data analysis. The water temperature was set at 25 °C. A platform (10 cm^2^), which was hidden from the mouse’s view, was located 2 cm beneath the surface of the water, allowing the mouse to easily climb onto it once its presence was detected. The MWM was placed in a well-lit white room with several posters and other distal visual stimuli hanging on the walls to provide spatial cues. The training on the hidden platform (spatial) version of the MWM was carried out on 4 consecutive days [26]. On each day, the mice (WT-ND, *n* = 8; WT-HFD, *n* = 10; TLR4-mut-ND, *n* = 10; TLR4-mut-HFD, *n* = 10) were allowed to swim for 60 s until they found the hidden platform. The time required to find the platform was recorded. On Day 5, a probe trial was conducted immediately after the four-day period to evaluate memory retention. The probe trial involved removing the submerged platform from the pool and allowing mice to swim for 60 s in any of the four quadrants of the pool. The time spent in the quadrant where the platform had been previously located was recorded. An observer blinded to the animals’ treatment group performed all the analyses.

### Analysis of body composition

Changes in body composition were determined by dual-energy X-ray absorptiometry (DEXA) using a densitometer (Lunar PIXImus; GE Medical Systems, Madison, WI, USA). This system employs a cone beam X-ray source generating energies of 35 and 80 keV and a flat 100 × 80 mm detector with individual pixel dimensions of 0.18 × 0.18 mm. The ratio of energy attenuation in the luminescent panel separates bone, lean tissue, and fat mass. A quality control procedure was routinely performed with a calibration phantom before imaging. Mice were anesthetized by intraperitoneal (IP) injection of 100 mg/kg ketamine and 10 mg/kg xylazine (Cristália, SP, Brazil) to ensure good immobilization and positioning during a five-minute acquisition [27]. Analyses were performed at baseline and at the end of the 24th week of diet, and the results were expressed as a percentage of body fat.

### Cerebral intravital microscopy with epi-illumination and fluorescence

At the end of the diet period, all the animals were randomly assigned for microcirculatory analysis. Brain microcirculation of all groups of animals was observed via cranial window imaging, and the functional capillary density, leukocyte–endothelium interactions, and microvascular endothelial function were evaluated by fluorescence intravital video microscopy as previously described [28]. Briefly, the animals were anesthetized with ketamine (90 mg/kg, i.p.) and xylazine (10 mg/kg, i.p.) and fixed in a stereotaxic frame. Then, their left parietal bones were exposed via midline skin incisions, and a 5-mm-diameter cranial window (between the coronal and lambdoid sutures) was created with a high-speed drill. The dura mater and arachnoid membranes were subsequently excised and withdrawn to expose the cerebral microcirculation, after which the cranial windows were suffused with artificial cerebrospinal fluid (132 mM NaCl, 2.95 mM KCl, 1.71 mM CaCl_2_, 0.64 mM MgCl_2_, 24.6 mM NaHCO_3_, 3.71 mM dextrose, and 6.7 mM urea, pH 7.4, at 37 °C). After the intravenous administration of 0.1 ml of 1% fluorescein isothiocyanate (FITC)-labeled dextran (molecular weight of 150,000 kDa; Sigma Chemical Co., St. Louis, MO, USA), the brain capillaries were observed with epi-illumination at 460 to 490 nm using a 520-nm emission filter, and the images were acquired under a 10 × ocular and 10 × objective lens on a Zeiss Axioscope A1 microscope (Zeiss, Oberkochen, Germany), producing a final magnification of 100 × on the monitor. The capillary count was performed using cellSens Standard 1.9 Zen blue software (Zeiss, Oberkochen, Germany), was used for one min per field. Only the continuously perfused capillaries were counted to determine the mean functional capillary density, expressed as the number of capillaries/mm^2^.

In addition, this cranial window enabled visualization of in vivo leukocyte recruitment. To label circulating leukocytes, 0.3 mg/kg rhodamine 6G was injected intravenously. Fluorescent leukocytes were visualized by intravital microscopy on postcapillary venules via epi-illumination at 510 to 550 nm using a 590-nm emission filter. The leukocyte–endothelial interaction was evaluated by counting the number of leukocytes adhering to the venular wall (100 µm long) over 30 s and was expressed as the number of cells/min/100 µm. Leukocyte rolling was defined as the movement of white blood cells into the vessel at a speed lower than the circulating red blood cells and was expressed as the number of cells/min. These parameters were determined in brain surface venules with diameters ranging from 50 to 120 cm. Microvascular endothelial function was evaluated by the pial arteriolar responses to the topical application of the endothelium-dependent vasodilator acetylcholine (ACh) in all animal groups (10^−6^ mol L^−1^). The cranial window was suffused with the vasoactive substance over 5 min, and the arteriolar diameters were measured before and after the application of ACh. Vascular responses were expressed as percent changes from the baseline.

### Tissue processing

After intravital microscopy, mice were euthanized by pentobarbital overdose (150 mg/kg, i.p.). The brain was dissected, and the left hemisphere cortex regions were stored in a freezer at − 80 °C for subsequent Western blot analysis.

For immunohistochemical and adipocyte analyses, 5 animals per group were anesthetized with ketamine and xylazine (90 and 10 mg/kg i.p., respectively), whole blood was collected for subsequent metabolic analyses, and the mice were transcardially perfused with 0.9% saline followed by 4% paraformaldehyde. The brains and epididymal white adipose tissue were collected and maintained in paraformaldehyde solution.

### Biochemical assays to evaluate metabolic function

The glucose test was carried out for whole blood using an automatic glucometer (One Touch Ultra 2®; Johnson & Johnson Medical S.A., Buenos Aires, Argentina). The blood was then centrifuged (15,000 rpm), and the serum was collected for determination of the insulin level by enzyme-linked immunosorbent assay (ELISA) using a commercial kit (Millipore, Burlington, MA, USA) following the manufacturer’s protocol. Insulin resistance was determined using the index of homeostasis model assessment (HOMA-IR) using the following formula [29]: HOMA-IR = fasting insulin (μU/ml) × fasting glucose (mmol/l)/22.5.

### Measurement of plasma concentrations of adipokines and metabolic hormones

Adipokines and metabolic hormones were measured in plasma that had been stored at − 80 °C after collection. The plasma concentrations of monocyte chemoattractant protein-1 (MCP-1), ghrelin, resistin, leptin, gastric inhibitory peptide (GIP) and glucagon were measured with a microbead-based Luminex multiplex immunoassay, the MILLIPLEX® MAP Kit for the Mouse Metabolic Hormone Expanded Panel (Millipore, Darmstadt, Germany), following the manufacturer’s protocol. These specific analytes were chosen for their effects on different (patho)physiological mechanisms: inflammation, glucose metabolism, and obesity-related hormones. All adipokine concentrations were reported in pg/ml.

### Adipocyte area quantification

Epididymal white adipose tissue sections were H&E stained and imaged at 40 × magnification (ZEISS Primo Star light microscopy Oberkochen, Germany). White adipocyte area was calculated using ImageJ software (NIH, Bethesda, MD, USA) by drawing ellipses circumscribing white adipocytes. The scale was set to 8 pixels per micron based on the pixel length of a 100-μm scale bar at 40 × magnification. Two to three images, each from a different area of a given sample, were captured per animal. Adipocyte area was measured for 10–20 adipocytes per image (25–40 adipocytes per mouse) and averaged on a per mouse basis.

### Western blot analysis

Protein levels of synaptophysin and postsynaptic density protein 95 (PSD-95) in the whole brain were determined by Western blot analysis. The brain was homogenized in 50 mM Tris, pH 8.0, containing 150 mM NaCl, 1 mM EDTA, 1% Triton X-100, 0.5% sodium deoxycholate, 0.1% SDS, and protease inhibitor cocktail (Complete Mini Protease Inhibitor Cocktail; Roche Diagnostics, Indianapolis, IN, USA). Homogenates were incubated for 30 min on ice, sonicated for 3 min, and centrifuged for 10 min at 10,000 × g and 4 °C. The supernatant was collected, and the protein concentration was measured using the bicinchoninic acid method with a BCA Protein Assay Kit (Thermo Scientific, Waltham, MA, USA), with bovine serum albumin (BSA) as the standard. Then, 50 μg of protein per lane was separated on a 12% SDS–PAGE gel and transferred to a nitrocellulose membrane (Bio-Rad Laboratories, Munich, Germany). After blocking in 5% nonfat milk in TBS-Tween-0.05% (blocking solution) for 2 h at room temperature, the membranes were incubated overnight at 4 °C with antibodies to synaptophysin (Abcam, ab8049, 1:000) and PSD-95 (Abcam, ab18258, 1:1000) in blocking solution. Then, TBS-Tween solution was used to wash the membrane in a shaker three times for 5 min per wash. After that, the membranes were incubated with the following secondary antibodies: horse antimouse IgG antibody (H + L), peroxidase (Vector Laboratories, PI-2000, 1:10,000), goat antirabbit IgG antibody (H + L), and peroxidase (Vector Laboratories, PI-1000, 1:10,000) in blocking solution for 1 h at room temperature. Then, the membranes were washed in a shaker three times for 5 min per wash followed by three times for 10 min per wash in TBS-Tween. Finally, using a hypercassette™ (Amersham Pharmacia Biotech, UK), the nitrocellulose membrane was exposed to the luminescent agent (A:B = 1:1) in a chemiluminescence film (Amersham Hyperfilm™, GE Healthcare Limited).

Beta-tubulin (Proteintech, 66240-1-Ig, 1:20,000) was used as a loading control. Densitometry analysis was performed by Odyssey software (Li-Cor Biosciences, Lincoln, NE, USA).

### Immunohistochemical analysis

For immunohistochemistry analysis, the brains were quickly excised after perfusion fixation as described above and serially sectioned at 50 μm. The sections were washed with PBS and incubated with 10% NGS diluted in PBS with 0.3% Triton X-100 for 90 min. The sections were then incubated with antibodies to glial fibrillary acidic protein (GFAP, Dako Glostrup, Denmark; 1:400), to ionized calcium-binding adaptor molecule 1 (Iba1, Wako; Tokyo, Japan 1:200), to TLR4 (MTS510; Santa Cruz Biotechnology, INC) or with biotinylated isolectin B4 (IB4; Vector Laboratories California, USA 1:100) overnight at 4 °C, washed again with PBS and incubated with secondary antibodies conjugated with Alexa Fluor 488 or 546 (Sigma–Aldrich Missouri, USA 1:400) or streptavidin-Cy3 (Sigma–Aldrich Missouri, USA 1:400) for 2 h. The sections were counterstained with DAPI, and coverslips were mounted with fluoromount (Vectashield—Vector Laboratories California, USA). Negative controls were treated with nonimmune rabbit IgG (Sigma–Aldrich Missouri, USA). Slices were imaged using a confocal microscope (Zeiss LSM 510 META; Zeiss, Oberkochen, Germany) equipped with a 63 × NA 1.40 oil immersion objective. The same selection criteria were carefully applied to all slices. The CA1 region of the hippocampus was first located followed by the parietal cortex.

For microglial morphology analysis, the ImageJ Sholl Analysis tool was used. This is a quantitative analysis method commonly used in neuronal studies to characterize the morphological characteristics of an imaged neuron. Using Sholl analysis, a mathematical algorithm of the program called the “branching index” is used to analyze neuronal morphology [30]. This index compares the difference in the number of intersections by making consecutive circles in relation to the distance of the neuronal sum. In this study, microglial processes were quantified as the number of intersections from 2 to 40 μm from the sum and the total number of intersections within the analysis circles, representing the total branches of the microglia.

To quantify morphological changes in the IBA1^+^ cells, consecutive Z-stack images were converted to a maximum-intensity projection image in ImageJ software (NIH, Bethesda, MD, USA). Using the Sholl analysis plugin, concentric circles were created, centered on the soma, beginning at 5.5-μm radii and increasing 2 μm with every circle. The number of intersections made by microglial branching processes with each successive increasing circle and the maximum number of intersections for the cell (Nm) were determined, as well as the critical value at which Nm occurred and the maximum length at which a branch intersection was observed [31].

For the analysis of the cerebral microvascular angioarchitecture, the AngioTool program was used (https://ccrod.cancer.gov/confluence/display/ROB2/Downloads) to determine the total length of the brain capillaries in each image obtained by confocal microscopy. AngioTool is a validated source for measuring vascular networks [32] and has already been described in analyses of murine brain and retinal angiogenesis [33].

Confocal images of vessels immunostained with IB4 isolectin were analyzed using the AngioTool program and were processed as described [34]. Images for quantitative analysis were acquired with a confocal microscope using a 20 × objective (oil) zoom (200 ×). The image was immediately segmented and skeletonized, and the analysis of the vasculature was performed automatically. The resulting image shows the overlay of the area of all vessels and the computed branching points inside the imaged area.

### Statistical analysis

The results are expressed as the mean ± standard error of the mean (SEM) for each group. Between-group statistical comparisons were performed using one-way analysis of variance (ANOVA), followed by Bonferroni’s post hoc test. When appropriate, the results were analyzed using unpaired Student’s *t* tests. Differences with *p* values less than 0.05 were considered significant. All calculations were performed using commercially available statistical software (GraphPad Prism, La Jolla, CA, USA).

## Results

### TLR4-mutant mice do not develop metabolic syndrome

Table 1 summarizes the average measures of the mice in the WT and TLR4-mut groups fed either a normolipid diet, ND, or a high fat diet, HFD. We observed marked increases in abdominal fat and plasma fasting glucose, as well as increased blood pressure, insulin levels, HOMA-IR, and cholesterol parameters, in the WT-HFD group compared to the WT-ND group. However, the TLR4-mut-HFD group did not present any significant difference in these parameters compared to the TLR4-mut-ND group. There were increases in cardiometabolic parameters in the TLR4 mutant mice fed a normal diet as compared to their WT controls; however, these increases were not statistically significant. In addition, the measures were also higher in the WT-HFD group than in the TLR4-mut-HFD group.

**Table 1:** Effects of high fat diet consumption on the cardiometabolic parameters of WT or TLR4-mut mice

Parameters	WT		TLR4-mut	
	ND	HFD	ND	HFD
Body weight (g)	38 ± 1	41 ± 0.8	36 ± 0.7	39 ± 0.6
Abdominal fat (g)	1.53 ± 0.1	2.47 ± 0.2**	1.7 ± 0.1	1.9 ± 0.1^#^
Body fat (%)	35.6 ± 1.4	47.2 ± 1.5**	37.5 ± 2.9	43.3 ± 1.2
SBP (mmHg)	94.5 ± 1.3	151.4 ± 2.4*	94.1 ± 2.5	98.7 ± 2.9^#^
Fasting glucose (mg/dl)	101.3 ± 13.5	170.9 ± 10.7**	122.7 ± 8.2	129.6 ± 7.5^#^
Fasting insulin (ng/ml)	0.21 ± 0.1	0.67 ± 0.3*	0.29 ± 0.7	0.26 ± 0.7^#^
HOMA-IR	1.51 ± 0.7	7.82 ± 3.7*	2.57 ± 0.7	2.43 ± 0.7^#^
Total cholesterol (mg/dl)	120.1 ± 18.1	180.2 ± 12.2*	140.4 ± 15.6	139.9 ± 6.3^#^
Triglycerides (mg/dl)	59.2 ± 7.9	264.6 ± 29.2***	47.2 ± 6.4	58.4 ± 4.7^###^

*SBP* systolic blood pressure, *HOMA-IR* homeostatic model assessment. Values represent the mean ± SEM of 8–12 animals per group. *p < 0.05, **p < 0.01, ***p < 0.001 vs. WT-ND, and ^#^*p* < 0.05 and ^###^*p* < 0.001 vs. WT-HFD

### TLR4 mutation mitigates adipocyte hypertrophy in the HFD-induced metabolic syndrome mouse model

**Fig. 1:**
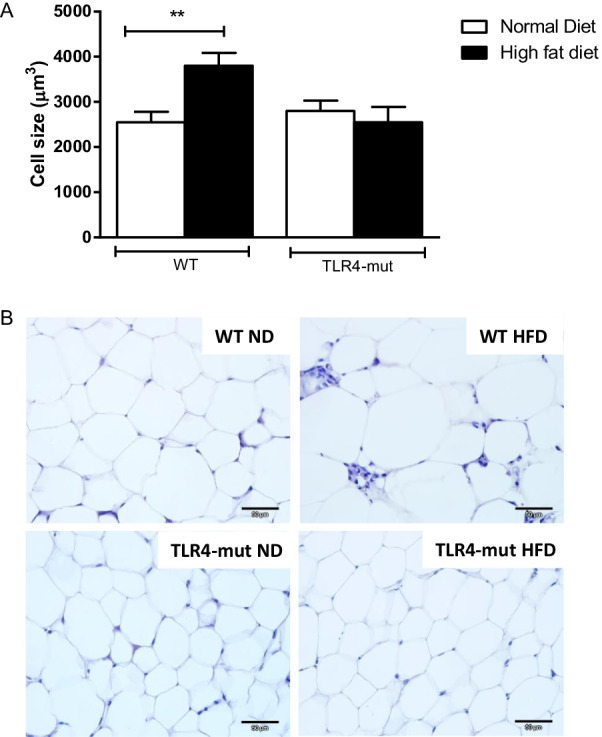
Quantitative analysis of size **A** and representative photomicrographs of epididymal adipocytes **B** from wild-type (WT) and TLR4-mut mice fed a normolipid diet (white bars) or a high-fat diet (black bars) for 24 weeks. The data are represented as the mean ± SEM, *n* = 6. Magnification 400x, scale bar 50 μm. **p < 0.01 vs. WT normal diet

The changes in adipocyte size in HFD-fed mice are shown in Fig. 1. WT-HFD mice had significantly larger adipocytes from the epididymal adipose tissue than all other groups. While the adipocytes of TLR4-mut-HFD mice were smaller than those of the WT-HFD group, there was no difference in the size compared to those of the WT-ND and TLR4-mut-ND mice (Fig. 1A, B).

In addition to significant differences in adipocyte size, histological examination indicated macrophage infiltration in the adipose tissue of WT-HFD mice but not in control mice (WT-ND) (Fig. 1A, B). Thus, we next decided to evaluate the levels of metabolic hormones in the plasma of these animals. Table 2 shows increased levels of ghrelin, and gastric inhibitory peptide (GIP), the leptin levels were increased but this were not statistically relevant. The glucagon levels decreased in the WT-HFD group compared with the WT-ND group. The TLR4-mut-HFD mice did not show any difference in the levels of these hormones when compared with the TLR4-mut-ND group.

**Table 2:** Effects of high fat diet consumption on the metabolic hormones of WT or TLR4-mut mice

Metabolic hormones (pg/mL)	WT		TLR4-mut	
	ND	HFD	ND	HFD
Leptin	2323 ± 166	3302 ± 407	1868 ± 777	2535 ± 312
Resistin	4730 ± 499	8649 ± 145	3221 ± 499	4495 ± 517^#^
Glucagon	11.9 ± 0.2	7.3 ± 0.9	6.5 ± 1.0	5.1 ± 0.7
Ghrelin	6.9 ± 0.3	11.2 ± 1.9*	8.5 ± 0.5	8.20 ± 0.8
GIP	220 ± 19	430 ± 134*	211 ± 44	237 ± 45

*GIP* glucose-dependent insulinotropic polypeptide. Values represent the mean ± SEM of 8–12 animals per group. *p < 0.05 vs. WT-ND and ^#^*p* < 0.05 versus WT-HFD

### TLR4 mutation prevents cerebral microcirculatory rarefaction and endothelial dysfunction in the brains of mice with HFD-induced metabolic syndrome

**Fig. 2:**
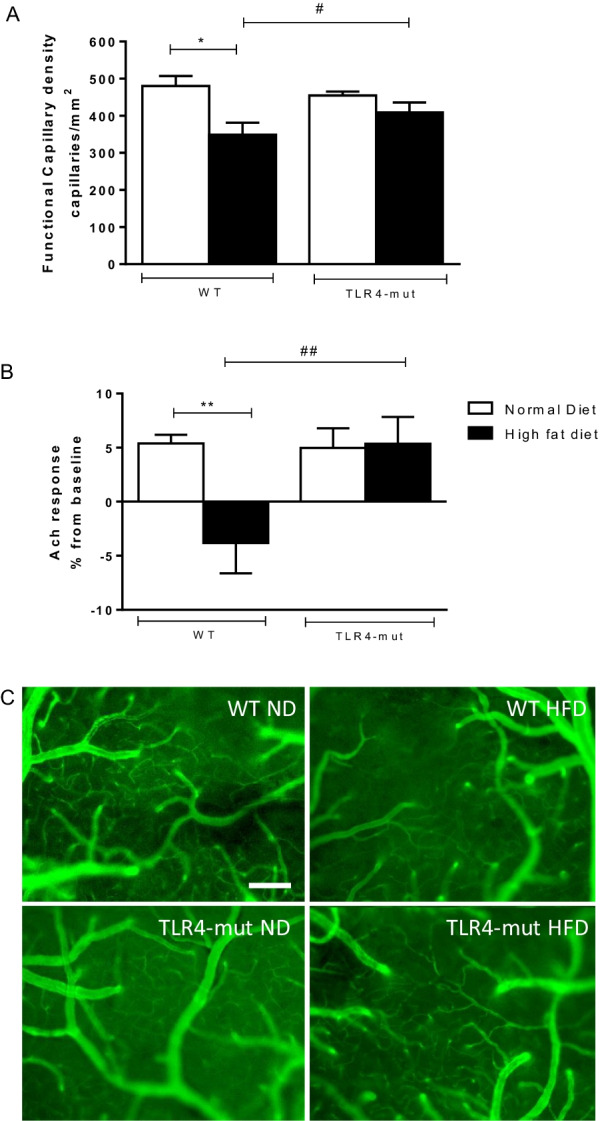
Brain microvascular functional capillary density (**A**). Evaluation of microvascular endothelial function **B** from wild-type (WT) and TLR4-mut mice fed a normolipid diet (white bars) or a high-fat diet (black bars) for 24 weeks. Representative intravital fluorescence microscopic images after intravenous administration of FITC-labeled dextran (C). Magnification 100×, scale bar 50 µm. Values represent the mean ± SEM. *n* = 8. *p < 0.05, **p < 0.01 versus WT normal diet ^#^*p* < 0.05, ^##^*p* < 0.01 vs. WT high-fat diet

To investigate the potential protective effects of TLR4 mutation in an experimental model of MS, we performed intravital microscopic examinations at the end of the diet period. Figure 2 shows the influence of 24 weeks of HFD ingestion on the brain microcirculation of WT and TLR4-mut mice. The TLR4-mut mice fed the HFD did not show differences in the numbers of spontaneously perfused capillaries in their brain cortices compared to those fed the ND. However, the WT mice with MS (WT-HFD) exhibited a lower number of spontaneously perfused capillaries than the WT control mice (WT-ND) (Fig. 2A and C).

Intravital assessments of cerebrovascular function revealed that microvascular vasodilator responses to ACh were blunted in the WT-HFD group compared with the WT-ND group, whereas vasodilator responses to ACh were restored in the TLR4-mut-HFD and TLR4-mut-ND groups (Fig. 2B).

### TLR4 mutation mitigates the recruitment of adherent leukocytes into the cerebral microvasculature of HFD-induced metabolic syndrome mice

**Fig. 3:**
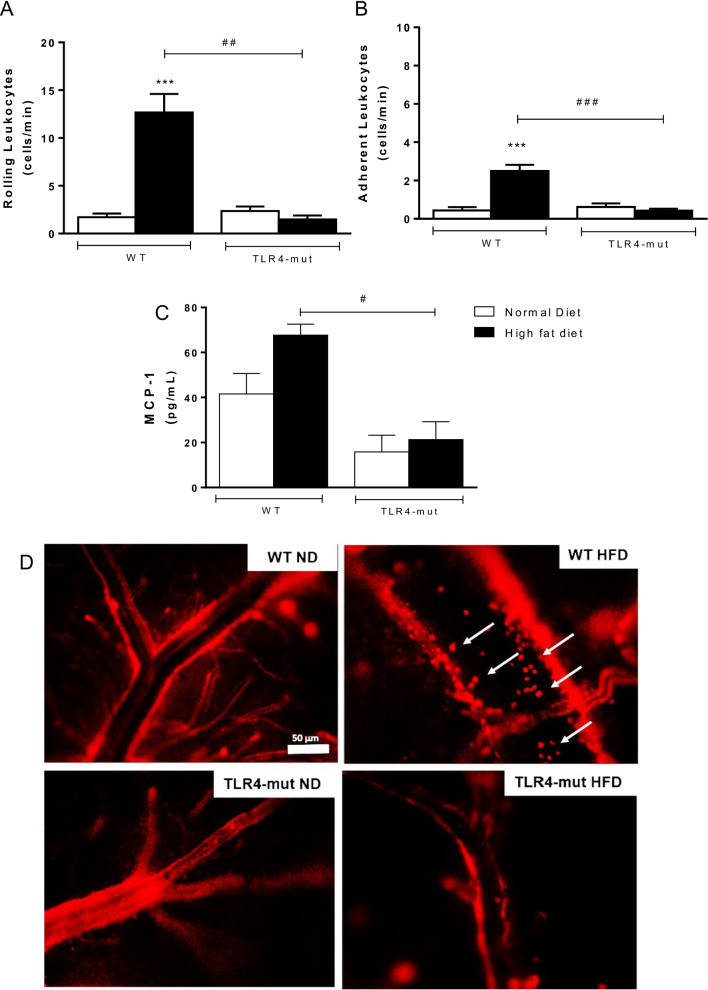
Leukocyte–endothelial interaction in the brain microcirculation. Rolling **A** and **B** adherent rhodamine 6G-labeled leukocytes evaluated by intravital fluorescence microscopy on the cerebral postcapillary venules, monocyte chemoattractant protein-1 (MCP-1) by multiplex immunoassay **C** and representative images of the brain microcirculation in the cerebral postcapillary venules, arrows indicate leukocytes **D** of wild-type (WT) and TLR4-mut mice fed a normolipid diet (white bars) or a high-fat diet (black bars) for 24 weeks. Magnification 200×, scale bar 50 µm. Values represent the mean ± SEM. *n* = 8 ***p < 0.001 vs. WT-normal diet, ^###^*p* < 0.001, versus WT high-fat diet

Intravital fluorescence microscopic examinations of the cerebral venules of WT-HFD mice showed marked increases in the numbers of rolling and firmly adherent leukocytes in comparison with those of the WT-ND group, while in the TLR4-mut-HFD group, this inflammatory event was not observed in the cerebral microvasculature (Fig. 3A, B and D). In addition, the levels of MCP-1, a potent chemotactic factor for monocytes, were significantly reduced in TLR4-mut-HFD mice compared to WT-HFD mice (Fig. 3C).

### TLR4 mutation protects animals from metabolic syndrome-associated cognitive decline

**Fig. 4:**
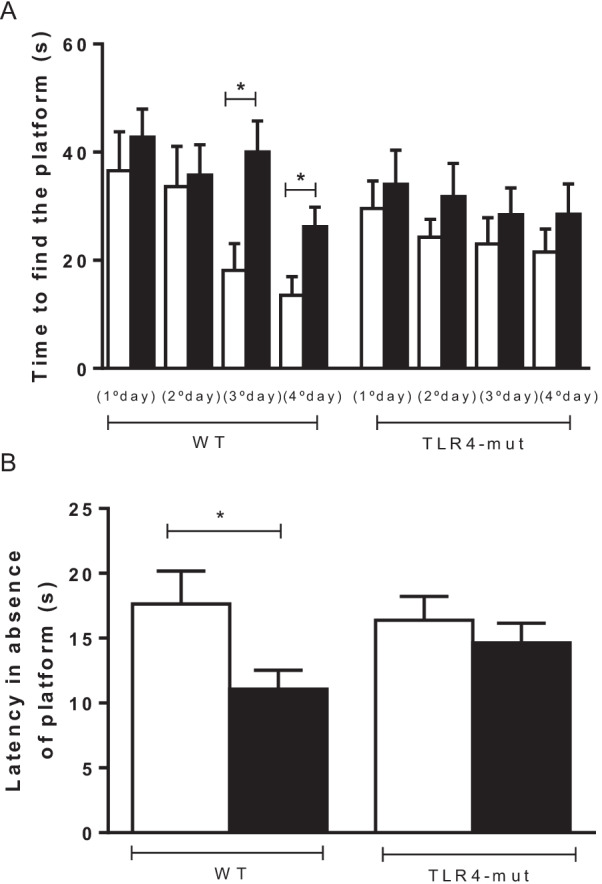
Spatial memory impairment **A** and time to find the platform, trial in the absence of the platform **B** by the MWM test of wild-type (WT) and TLR4-mut mice fed a normolipid diet (white bars) or a high-fat diet (black bars) for 24 weeks. The data are expressed as the mean ± SEM *p < 0.05, *n* = 8–15/group

Figure 4 shows the effect of HFD-induced MS on the cognitive performance of WT and TLR4-mut mice, as assessed by the MWM test. WT-HFD mice took longer to find the platform from the third day (Fig. 4A) and spent a shorter time in the quadrant from which the platform was removed (Fig. 4B) than mice from the WT-ND group. TLR4-mut mice fed both the HFD and ND performed similarly in both tasks (Fig. 4), indicating that the TLR4 mutation protected animals from MS-associated cognitive decline.

### TLR4 mutation restored synaptic protein expression in the brains of HFD-induced metabolic syndrome mice

**Fig. 5:**
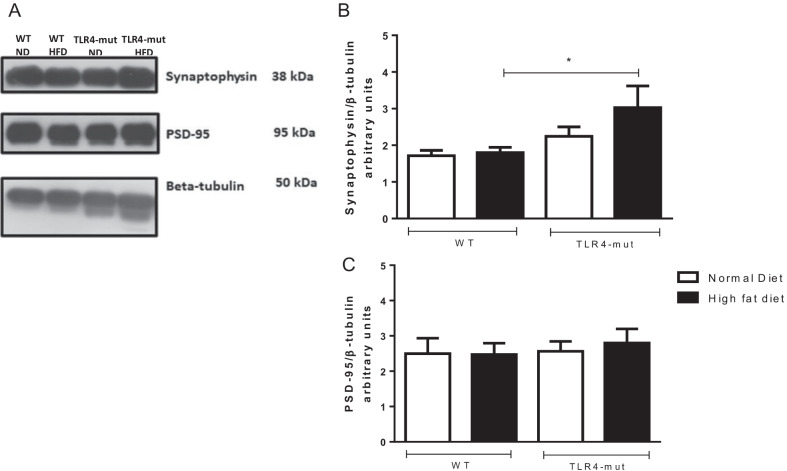
Western blot analysis **A** and quantification of the expression of synaptophysin **B** and PSD-95 **C** in the whole brain from wild-type (WT) and TLR4-mut mice fed a normolipid diet (white bars) or a high-fat diet (black bars) for 24 weeks. Values represent the mean ± SEM of 4–7 animals per group. Quantitative protein expression of synaptophysin and PSD-95 bands detected by western blot using beta-tubulin as a loading control. *p < 0.05 versus WT-HFD

The expression levels of proteins related to brain plasticity, synaptophysin and PSD-95, were evaluated by Western blot analysis of the whole brain. The HFD group of TLR4-mut mice showed a significant increase in synaptophysin compared to the WT-HFD group. However, there was no difference in the expression of PSD-95 in the TLR4-mut HFD group compared to the WT-HFD group (Fig. 5).

### TLR4 mutation improves astrocyte interaction with the hippocampal vasculature in HFD-induced metabolic syndrome mice

**Fig. 6:**
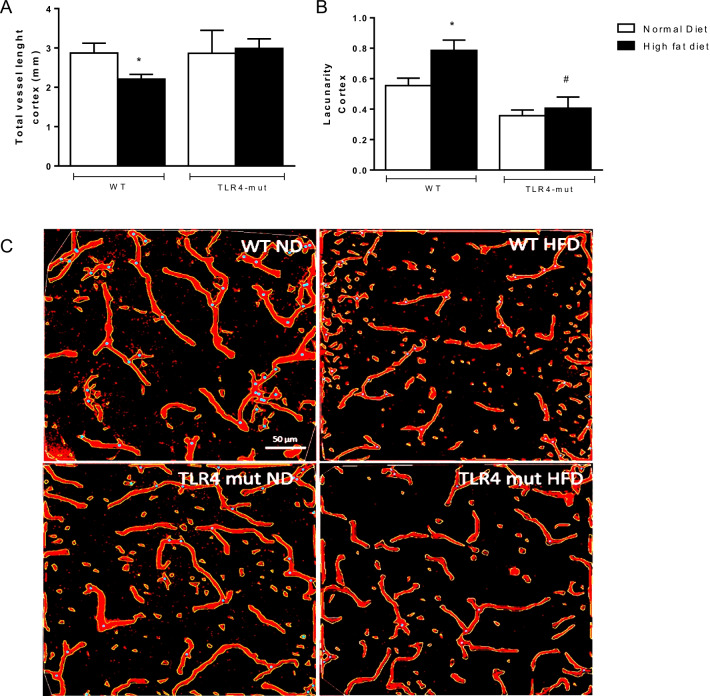
Graphical representation of the AngioTool analysis in the cerebral cortex. Total vessel length **A** and lacunarity (**B**). The results of AngioTool analysis of confocal images of brain slices of cortex from wild type (WT) and TLR4-mut mice fed a normolipid diet (white bars) or a high-fat diet (black bars) for 24 weeks. Skeleton in red and branching points in blue. Vessels labeled with IB4, scale bar 50 µm. *n* = 6. *p < 0.05 versus WT-ND and ^#^*p* < 0.05 versus WT-HFD

Next, we examined brain vessel morphology, including astrocytes, microglia, and neurons. Figure 6 shows the quantitative results, with the corresponding segmented cortex images obtained from AngioToll software. Measurements of the following parameters were obtained: the total vessel length and the lacunarity. We observed a significant decrease in the total vessel length (*p* < 0.05) in mice of the WT-HFD group, while the lacunarity was higher than that in the WT-ND group. However, in the TLR4-mut-HFD group, there was no difference observed in either of these parameters.

We also examined the hippocampus, a region of the brain responsible for consolidating short- and long-term memory [35], which has been shown to be particularly prone to inflammation and damage during metabolic disease [36]. However, no alterations in the total vessel length or lacunarity were noticed in the WT-HFD and TLR4-mut-HFD groups when compared with their respective ND controls (data not shown).

**Fig. 7:**
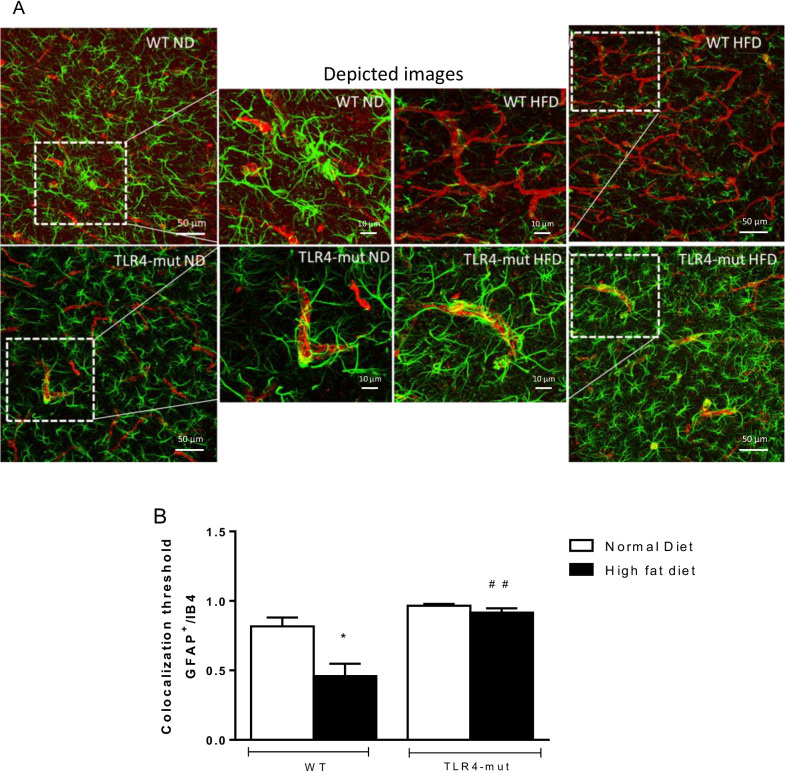
Colocalization of astrocytes with hippocampal vessels. **A** Confocal microscopic images of hippocampal sections marked with anti-IB4 for endothelium (orange), anti-GFAP (green) for astrocytes and DAPI (blue) for wild-type (WT) and TLR4-mut mice fed a normolipid diet (white bars) or a high-fat diet (black bars) for 24 weeks. The enlarged images show examples of contact between the immunoreactive astrocytic processes and IB4 of the vessel. Magnification: 400x, scale bar 50 µm and 10 µm in depicted pictures. **B** Graphical representation of the colocalization of GFAP/IB4 *p < 0.05 versus WT-ND ^##^*p* < 0.01 vs. WT-HFD. *n* = 5

Astrocytes and microglia and their interaction with the hippocampal vasculature are important features of NVU and blood–brain barrier (BBB) integrity (21). Using immunofluorescence, we quantified the numbers of Iba1^+^ microglia and assessed the area of astrocytes as denoted by GFAP^+^ staining. In analysis of colocalization of astrocytes and vessels in cerebral microcirculation (Fig. 7A), the coverage with astrocyte endfeet in the WT-HFD group was markedly decreased compared to that in the WT-ND group; however, in the TLR4-mut-HFD group we observed the same colocalization values as compared to the TLR4-mut-ND group (Fig. 7B).

### HFD-induced metabolic syndrome changes the microglial morphology

**Fig. 8:**
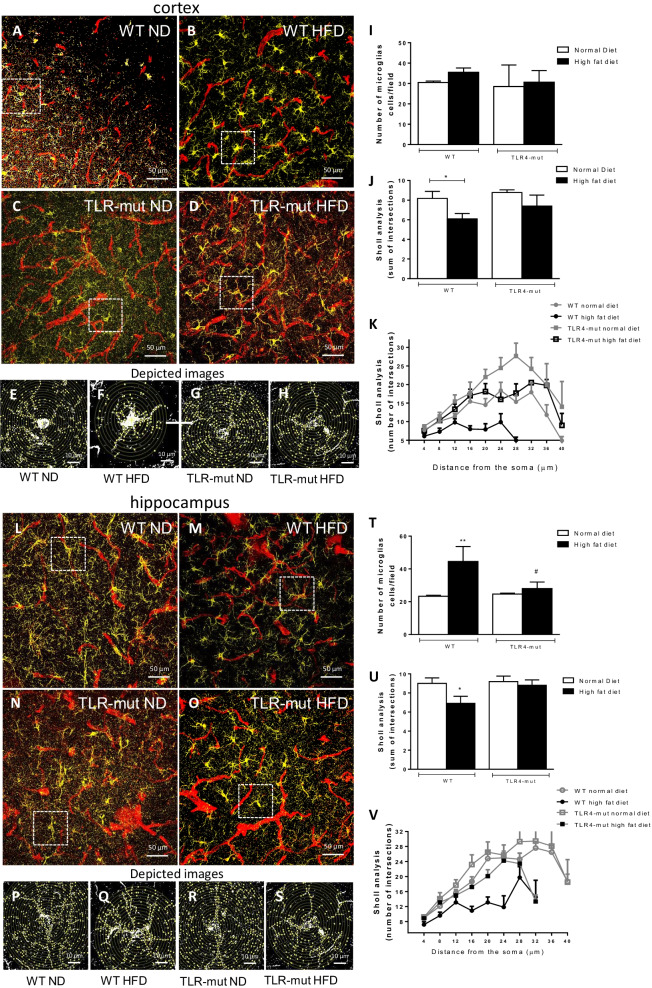
Analysis of microglia. Confocal microscopic images of cortex sections (upper panel: **A**–**D**) and hippocampus (bottom panel: L-O) with a single cell of each image cropped to show details of Sholl analysis in cortex (upper panel: **E**–**H**) and hippocampus (bottom panel: **P**–**S**). Samples marked with anti-Iba1 for microglia (yellow) and anti-IB4 (red) for vessels of wild type (WT) and TLR4-mut mice fed a normolipid diet (white bars) or a high-fat diet (black bars) for 24 weeks. The data represent the mean ± SEM of up to 40 microglial cells per group of 3 animals. Magnification 400x, scale bar 50 µm and 10 µm in depicted pictures. Graphs of the number of microglia/field **I** and **T** and Sholl analysis (**J**, **K**, **U**, and **V**) * p < 0.05 versus WT-ND, **p < 0.01 vs. WT-ND ^#^*p* < 0.05 versus WT-HFD

We decided to examine microglial morphology in cortex and hippocampus of all groups of animals. We observed that the microglia of WT-HFD mice presented an amoeboid phenotype, with retracted processes as shown in Fig. 8 (in cortex: B and F; in hippocampus: M and Q), while the WT-ND group presented more ramified microglial morphology in sections of the cortex (Fig. 8A and E) and the hippocampus (Fig. 8L and P). This is supported by our quantitative data, as the maximum number of hippocampal microglia increased in WT-HFD (Fig. 8; T), and the number of branches decreased in both cortical (Fig. 8J and K) and hippocampal sections (Fig. 8U and V) compared to the WT-ND group. No difference was observed in the TLR4-mut-HFD group in either the cortex (Fig. 8D and H) or the hippocampus (Fig. 8O and S) compared to the TLR4-mut ND group in the cortex (Fig. 8C and G) or hippocampus (Fig. 8N and R), suggesting that microglial activation is an indicator of inflammation after MS and it is mitigated by TLR4 mutation (Fig. 8T–V).

## Discussion

Studies of human and animal models provide strong evidence that alterations in glucose metabolism, such as hyperglycemia or MS, could have a negative effect on brain function [29, 37, 38]. In this study, we explored the specific contributions of a HFD to neuroinflammation in an experimental mouse model of MS. We demonstrate that long-term ingestion of a HFD induces region-specific effects on the brain, elevating microglial numbers, and decreasing the coverage of astrocytes in the cerebral vessels of the hippocampus. Moreover, we provide evidence that the ingestion of a HFD led to increased fasting blood glucose and abdominal fat, which are features of MS also observed in patients [39, 40]. Visceral adiposity plays a key role in the development of human MS and is associated with all MS criteria [41]. The increase in visceral adipose tissue leads to adipocyte hypertrophy, which is considered a key event in insulin resistance in obesity and MS, as it not only elevates proinflammatory cytokine levels but also leads to leptin resistance [42–44]. We demonstrated that HFD intake in the WT group led to higher visceral fat accumulation that was not observed in TLR4 mutant mice; we also demonstrated in this study that a mutation in the TLR4 receptor reduces leukocyte–endothelium interactions, particularly the numbers of rolling and adherent leukocytes in the cerebral microvasculature. Moreover, TLR4 appears to contribute to the development of regional neuroinflammation in animal models of MS. We confirmed the upregulation expression of TLR4 in MS by immunostaining TLR4 in the brain cortex of the WT-HFD group as compared to the WT-ND group, and it was possible to correlate this increased expression with the observed metabolic syndrome-related changes. These results are shown in the Additional file 1.

An attenuation of obesity-induced adipose tissue inflammation has already been demonstrated in C3H/HeJ mice (which have a mutation in TLR4) when compared to their controls after ingestion of a HFD [24, 45]. TLR4 dysfunction in these animals suggests a fundamental role of this receptor in the diet-induced hyperlipidic inflammatory response [11]. In fact, there were increases in cardiometabolic parameters in the TLR4 mutant mice fed a normal diet compared to their WT controls; however, these increases were not statistically significant. In addition, it is important to emphasize that even with the nonsignificant increase in cardiometabolic parameters, this was not able to generate an inflammatory effect, reinforcing the importance in TLR4 as mediator of this process.

Saturated fatty acids (SFAs) can trigger inflammatory pathways, including those mediated by TLR4, similar to lipopolysaccharides (LPS) [46, 47] [48]. We believe that the source of fatty acids from the HFD used in the present study may be an important proinflammatory factor in the induction of MS. Indeed, some studies have shown that TLR4 activation in visceral adipose tissue is important in the genesis of the low-grade chronic inflammatory response observed in MS [11, 49, 50]. Once activated, these pathways can increase the synthesis and secretion of many chemokines (e.g., MCP-1) in adipocytes, contributing to insulin resistance and proinflammatory macrophage infiltration [50].

In the present work, we evaluated adipocytokines (insulin, leptin, resistin) and gut hormones (ghrelin and GIP). We observed increased levels of adipocytokines in the WT-HFD group compared to the WT-ND group but not in the TLR4-mut-HFD group, suggesting that this receptor may have an important role in insulin and leptin resistance in MS. Consistent with these findings, other studies have demonstrated that the deletion, knockdown or pharmacological inhibition of TLR4 protects mice from leptin and insulin resistance [45, 51].

An increase in resistin levels has been observed in obese patients compared to those with normal body mass. However, ghrelin, an appetite-regulating hormone secreted during fasting by gastrointestinal endocrine cells, plays an important role in weight regulation [52]. Increased levels of serum ghrelin concentration have been observed in pathological conditions, including insulin resistance and diabetes, whereas physical exercise and weight loss can reduce blood levels of this hormone [53]. We also observed increased levels of ghrelin and GIP in the WT-HFD group compared with the WT-ND group but not in the TLR4-mut-HFD group, again suggesting that TLR4 triggers metabolic and hormonal alterations leading to MS. The increase in resistin levels observed in TLR4-mut HFD mice was significantly inferior when compared to WT HFD mice. This finding demonstrates that TLR4 is involved in the inflammation process caused by resistin release by adipose tissue. This result corroborates previous studies showing the role of resistin on the activation of TLR4 and may explain the spectrum of resistin effects beyond inflammation and a loss of TLR4 function preventing diet-induced obesity and insulin resistance [32]

Endothelial dysfunction is an important component of MS and is caused by an imbalance between vasodilatory substances such as nitric oxide (˚NO), vasoconstricting substances such as endothelin, and prothrombotic factors such as plasminogen-1 activator inhibitors [54]. The decrease in ˚NO levels in individuals with MS is already well established [55]. In the physiological state, insulin stimulates the production of ˚NO by endothelial cells through an increase in endothelial nitric oxide synthase (eNOS) activity. This action is reduced or overturned in the case of insulin resistance, as is seen in MS [56]. In the present study, the vasodilatory response to acetylcholine evaluated in superficial arterioles of the cerebral cortex was abolished after the HFD period in the WT group but not in the TLR-mut group. Studies have already shown that 12 weeks of eating a HFD is sufficient to induce endothelial dysfunction in the cerebral microvascular bed [57]. Moreover, TLR4 contributes to endothelial dysfunction observed in hypertension, and anti-TLR4 treatment improves endothelium-dependent relaxation [58]. Our study also shows that HFD consumption induces functional capillary rarefaction in the brain, accompanied by increased vascular inflammation characterized by significant amounts of leukocyte rolling and adhesion to venular walls.

Several studies have reported that MS is increasingly related to cognitive decline [59–62]. The reduction in cerebral blood flow is responsible for lower cognitive performance due to neuronal damage in brain regions related to memory and learning [63]. We employed the Morris water maze test, a complex task that assesses not only hippocampus-dependent reference memory changes but also spatial working memory and working memory load. Our results demonstrate that TLR4-mut mice, regardless of diet, learned the task, exhibiting a learning curve and a decreasing number of errors during the test. Conversely, the WT-HFD group exhibited worse performance on the Morris water maze test, indicating that the decline in cognitive function is prevented in TLR4 mutant mice.

The NVU includes neurons, astrocytes, pericytes, and microglia, as well as the blood vessels themselves, and enables tight regulation of blood flow through the brain vasculature [20]. Astrocytes support neuronal function by providing essential structural and nutritional support. As astrocytes are involved in neurotransmitter trafficking, they are also sensitive to circulating endocrine signals, such as the hormones ghrelin, glucagon‐like peptide‐1, and leptin, which have major impacts on central nervous system (CNS) mechanisms, controlling food intake and energy balance [64, 65]. Therefore, any disruption to astrocyte endfeet can have dramatic consequences on the integrity of the BBB, with consequent development of brain disorders [65]. In the present work, we used immunohistochemistry to assess the coverage of astrocytes in brain microvessels. Our results showed that the coverage of cerebral microvessels by astrocyte endfeet was markedly decreased in WT animals with MS compared to their controls; however, the TLR4 mutation appears to prevent this interruption in the endfeet coverage after chronic ingestion of the HFD.

To investigate whether differences in the neuronal and glial components could account for the diet-related changes found in the cognitive tests, brain cortical and hippocampal microglia morphology were analyzed. Microglia appear to play a key role in the inflammatory response that triggers neuronal death and synaptic dysfunction. Changes in microglial phenotype were observed, suggesting activated microglia in the cortex and hippocampus of WT-HFD mice that might indicate a greater degree of inflammation [32]. However, neither the HFD nor the ND TLR4-mut mice presented changes in microglial morphology, reinforcing our interpretation that TLR4 is a key player in the inflammatory component in MS. Under steady-state conditions, the number and function of microglia are tightly regulated, comprising the immune dominant cell type in the brain. In response to an acute immunological stimulus, microglia proliferate, increase phagocytosis, and eliminate pathogenic insult and later undergo apoptosis after the resolution of inflammation. In the case of a chronic stimulus, such as low-grade inflammation, characteristic of metabolic syndrome, microglia can become activated and remarkably long lasting, releasing cytokines and contributing to the perpetuation of a harmful environment [66]. In our study, microglia activation could be inferred by sholl analysis, which is considered the gold standard for studying microglial morphology and a good marker for an inflammatory response [67]; Furthermore, Intravital microscopy analysis in the brain was also performed to assess leukocyte–endothelium interactions, particularly the number of rolling and adherent leukocytes in the brain microvasculature. Rolling and adhesion of leukocytes were both increased in HFD animals and are regarded as classic and well characterized early events of the inflammatory process associated with both endothelial (with expression of adhesion molecules) and leukocyte activation. Finally, secretion of chemical mediators such as cytokines and chemokines are another hallmark of an inflammatory response, and in that sense, we showed elevated levels of monocyte chemoattractant protein-1 (MCP-1) HFD animals. However, a full inflammatory process is not undoubtedly demonstrated in our study. In addition, it is reasonable to assume that inflammation under the conditions of our study shall be low grade, not exuberant, and long lasting.

It has been suggested that prolonged feeding with a high-fat and carbohydrate-rich diet reduces neuroplasticity processes, as well as hippocampal innervation, which is also associated with cognitive impairment [68]. Neuronal plasticity reflects the ability of synapses to be modified in number and strength in response to physiological and pathological stimuli. We analyzed the expression levels of neurotrophic factors, such as synaptophysin, a presynaptic marker, and PSD-95, a postsynaptic marker. We wanted to understand the putative protective effect of the TLR4 mutation on neuronal plasticity in an HFD MS-induced model. Synapse-associated proteins, such as the postsynaptic density 95 (PSD-95) and synaptophysin, are essential for synaptic maturation, plasticity, learning, and cognition. [69–71]. PSD-95 is a member of a family of proteins known as membrane-associated guanylate kinases [72] and the most abundant protein at the postsynaptic density [73]. In the same way, synaptophysin is another synaptic marker protein, located in the synaptic vesicle membrane and its increase indicates greater synaptic activity [74]. An increase in synaptophysin and PSD-95 occurs during the first decade of life, highlighting the critical role of these proteins in synaptogenesis [75]. It is worth noting that synaptic dysregulation is observed in neuroinflammation and neurological disorders, demonstrating a correlation between synaptic loss and cognitive impairments [76, 77]. In addition, therapeutic approaches that are able to induce and improve the expression of these proteins are associated with neuroprotection and improvement of cognitive function and memory [78, 79].

We observed that mice in the TLR4-mut group showed an increase in synaptophysin protein content, but this did not occur in the WT-HFD group. However, no significant alterations were observed in the PSD-95 protein content in either group. Several studies have already demonstrated that long-term consumption of HFD may be activating signaling pathways with deleterious effects in various regions of the brain [80, 81]. It is known that differences may occur in the synaptic activity of brain regions, such as the cortex and the hippocampus, the latter being the memory storage center and an important region for the memory of habituation to a new environment. We believe that no differences were observed in PSD-95, as the analyses were performed in the whole brain and not in specific brain regions, and we consider this one limitation of this study. Therefore, the results suggest that although the expression of synaptic proteins are not altered, the reduction in the astrocytic–vessel communication can greatly compromise the integrity of the NVU and, consequently, the cognitive function of the animals.

The relationship between MS and neurodegeneration has many unanswered questions. There is consensus that inflammation is an inherent problem in this syndrome, but it is still unclear whether a component of the high-fat diet is primarily responsible for inducing inflammation or whether the inflammatory profile of visceral adipose tissue is a critical trigger of this process. Furthermore, evaluating the local modulation of TLR4 would be an interesting pharmacological approach to prevent or treat cognitive decline in MS and could be the subject of future studies.

## Conclusions

Our data suggest that TLR4 signaling activation is involved in microvascular dysfunction and neuroinflammation associated with HFD-induced MS and that this receptor possibly plays a causative role in the development of cognitive decline.

## Supplementary Information

**Additional file 1. **Representative Toll LikeReceptor (TLR4) immunofluorescence staining of samples from the cortex of mice fed on a high-fat diet (HFD) oron normolipid diet (ND) for 24 weeks. Magnification ×200 (scale bar, 100 μm), nuclei are stained in blue by DAPI,and TLR4 are stained in green by FITC (with the flatten overlay tool of ImageJ software). Graphical representationof the TLR4^+^ cells (A) and the fluorescence intensity (B). Data represent the mean ± SEM *p < 0,05 and **p < 0,01,versus ND; n = 4.

## Data Availability

The datasets used and/or analyzed during the current study are available from the corresponding author on reasonable request.

## Abbreviations

ACh: Acetylcholine
BBB: Blood–brain barrier
BDNF: Neurotrophic factors derived from the brain
DEXA: Dual-energy X-ray absorptiometry
eNOS: Endothelial nitric oxide synthase
FITC: Fluorescein isothiocyanate
GFAP: Glial fibrillary acidic protein
GIP: Glucose-dependent insulinotropic polypeptide
HFD: High-fat diet
HR: Heart rate
IB4: Biotinylated isolectin B4
Iba1: Ionized calcium-binding adaptor molecule 1
LPS: Lipopolysaccharides
MCP-1: Monocyte chemoattractant protein-1
MS: Metabolic syndrome
MWM test: Morris water maze test
ND: Normal diet
NVU: Neurovascular unit
PSD-95: Postsynaptic density protein 95
SAP: Systolic arterial pressure
SFAs: Saturated fatty acids
TLR4: Toll-like 4 receptor
TLRs: Toll-like receptors
