# Supplementary figures and images for: TLR4 mutation protects neurovascular function and cognitive decline in high-fat diet-fed mice
Source: J Neuroinflammation. 2022 Apr 29;19:104. doi: 10.1186/s12974-022-02465-3 (PMC9052472; doi:10.1186/s12974-022-02465-3)

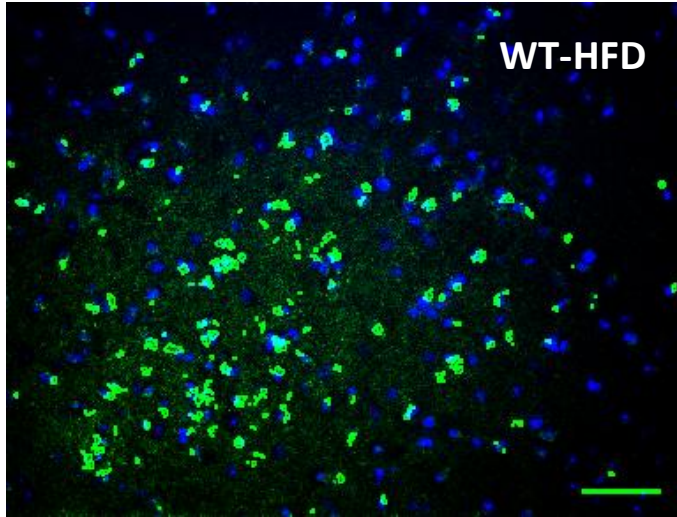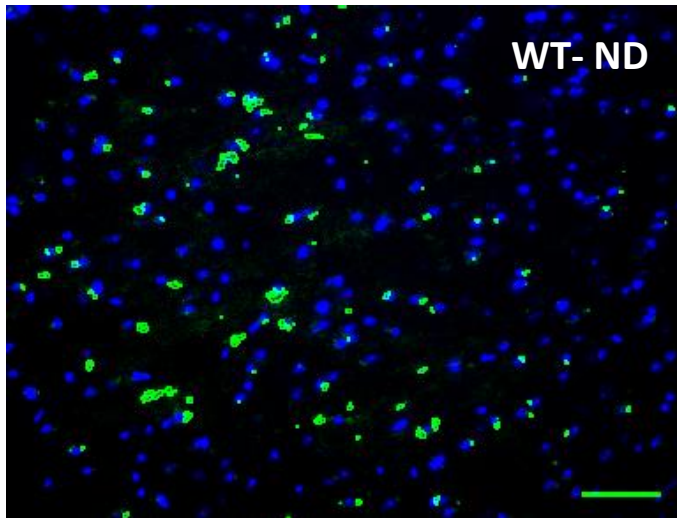

**A**

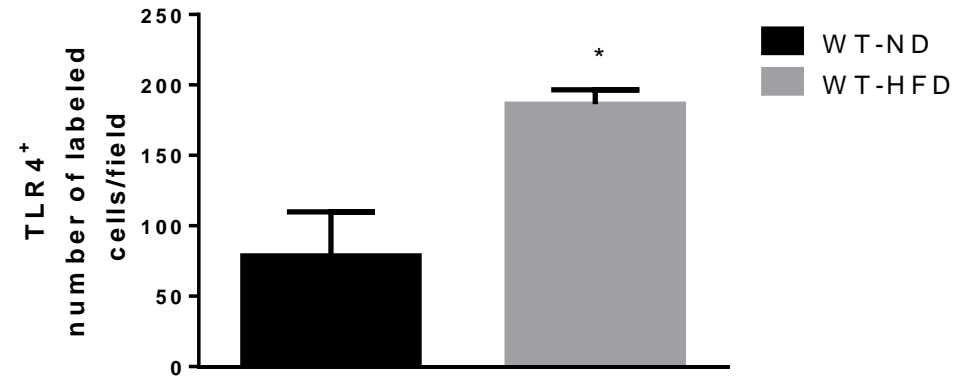

**B**

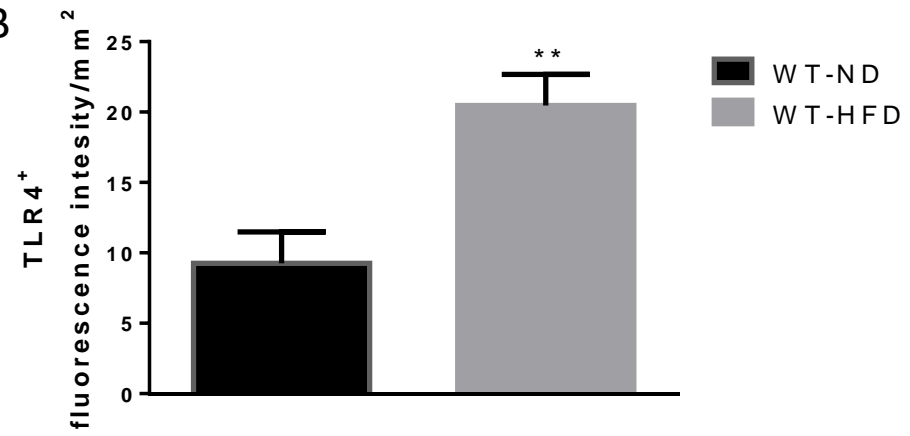

Supplement: Supplementary file 1 — Additional file 1. Representative Toll LikeReceptor (TLR4) immunofluorescence staining of samples from the cortex of mice fed on a high-fat diet (HFD) oron normolipid diet (ND) for 24 weeks. Magnification ×200 (scale bar, 100 μm), nuclei are stained in blue by DAPI,and TLR4 are stained in green by FITC (with the flatten overlay tool of ImageJ software). Graphical representationof the TLR4+ cells (A) and the fluorescence intensity (B). Data represent the mean ± SEM *p < 0,05 and **p < 0,01,versus ND; n = 4. [file 12974_2022_2465_MOESM1_ESM.pdf]
